# Association of lineage 4.2.2 of *Mycobacterium tuberculosis* with the 63-bp deletion variant of the *mpt64* gene

**DOI:** 10.1128/spectrum.01842-23

**Published:** 2023-11-10

**Authors:** Zexuan Song, Wencong He, Shaojun Pei, Bing Zhao, Xiaolong Cao, Yiting Wang, Ping He, Dongxin Liu, Aijing Ma, Xichao Ou, Hui Xia, Shengfen Wang, Chunfa Liu, Yanlin Zhao

**Affiliations:** 1 National Institute for Communicable Disease Control and Prevention, Chinese Center for Disease Control and Prevention, Beijing, China; 2 National Tuberculosis Reference Laboratory, Chinese Center for Disease Control and Prevention, Beijing, China; 3 School of Public Health, Peking University, Beijing, China; 4 Animal Science and Technology College, Beijing University of Agriculture, Beijing, China; Laboratory Corporation of America Holdings, Burlington, North Carolina, USA

**Keywords:** *Mycobacterium tuberculosis*, MPT64 antigen test, *mpt64*, lineage L4.2.2

## Abstract

Tuberculosis (TB) remains the primary cause of death from an infectious disease worldwide. The MPT64 antigen assay is an expedited diagnostic method that is increasingly adopted in many TB diagnostic laboratories and clinical settings worldwide. However, the diagnostic accuracy of the MPT64 antigen assay is influenced by the presence of 63bp deletion variants within the *mpt64* gene, and the potential association of this variant with the lineage or sub-lineage of *Mycobacterium tuberculosis* complex (MTBC) remains unverified. In this study, we analyzed the MPT64 antigen assay using isolates from the major lineage and sub-lineage of MTBC combined with whole-genome sequencing. Notably, we found a significant association between the 63bp deletion variant in the *mpt64* gene and *M. tuberculosis* Lineage 4.2.2 (L4.2.2) globally. Phylogenetic analysis revealed that L4.2.2 isolates were divided into two clusters (Clades A and B). Importantly, all isolates within Clade A exhibited the 63bp deletion variant in the *mpt64* gene. Furthermore, using the Bayesian Skyline population model, we observed that the effective population size of Clade A has remained relatively constant. These findings highlight that MPT64-based testing should be cautiously used, and MPT64 negative results should be validated using an alternative assay in regions where L4.2.2 isolates are prevalent, such as China and Vietnam. These L4.2.2 isolates with the 63bp deletion variant in *mpt64* warrant greater consideration during the development of diagnostic tests and study of their immunomodulatory role. Overall, this study provides valuable insights into a better understanding of the virulence and diagnosis of MTBC isolates based on the MPT64 protein.

**IMPORTANCE:**

To date, rapid diagnostic methods based on the MPT64 antigen assay are increasingly utilized to differentiate between non-tuberculous mycobacteria and TB disease in clinical settings. Furthermore, numerous novel techniques based on the MPT64 release assay are continuously being developed and applied for the identification of both pulmonary and extrapulmonary TB. However, the diagnostic accuracy of the MPT64 antigen assay is influenced by the presence of 63 bp deletion variants within the *mpt64* gene. To our knowledge, this is the first report on the association between the 63 bp deletion variant in *mpt64* and *Mycobacterium tuberculosis* L4.2.2 globally, which highlights the need for the cautious utilization of MPT64-based testing in regions where L4.2.2 isolates are prevalent, such as China and Vietnam, and MPT64 negative results should be confirmed with another assay. In addition, further studies on vaccine development and immunology based on MPT64 should consider these isolates with 63 bp deletion variant.

## INTRODUCTION

Tuberculosis (TB), caused by *Mycobacterium tuberculosis* (MTB), remains a major public health concern with a substantial disease burden. Timely and accurate TB diagnosis is crucial for preventing its transmission and for global TB management and eradication (1). Although the World Health Organization recommends the nucleic acid amplification test, known as Xpert MTB/RIF, for TB and rifampicin resistance identification, the traditional culture method remains the “gold standard” for diagnostic purposes. Acid-fast bacilli microscopic detection has traditionally been the primary TB diagnostic method in clinical settings. However, this method lacks the ability to distinguish between *Mycobacterium tuberculosis* complex (MTBC) and non-tuberculous mycobacteria (NTM). Recent years have witnessed an increase in pulmonary diseases brought on by NTM infections (2, 3). Given the different treatment approaches for MTBC and NTM infections regarding antimicrobial therapy, infection control measures, and clinical management strategies, it is imperative to differentiate between MTBC and NTM in diagnostic procedures.

MPT64, a 24KD protein secreted by MTBC during bacterial growth, is encoded by the *mpt64* gene (*Rv1980c*), a component of the region of difference two locus. MPT64 is frequently employed as a candidate protein for TB diagnosis and vaccine development, primarily due to its high specificity for MTBC (4). Presently, rapid diagnostics based on the MPT64 antigen assay are increasingly being used in numerous TB diagnostic laboratories worldwide (5, 6). The MPT64 protein is one of the biomarkers with significantly elevated expression in active TB patients, and numerous novel techniques for MPT64 release assay are constantly being developed and applied for the detection of pulmonary and extrapulmonary tuberculosis in clinical settings (4, 7, 8). Additionally, the MPT64 test has been utilized to distinguish between NTM and MTB infections in clinical settings (6, 9). Given its simplicity and speed, the MPT64 release assay holds promise as a useful alternative to the labor-intensive and time-consuming phenotypic approaches currently employed for treatment monitoring (4).

Nevertheless, previous studies have found that the MPT64 test exhibits reduced sensitivity in detecting MTB isolates of Lineages 5 and 6 (5, 10). Genetic polymorphisms in the *mpt64* gene can impact the MPT64 antigen structure, leading to false-negative results in MPT64-based assays, particularly for the 63 bp deletion (amino acid positions 66–86) (11). To date, diagnostic approaches based on the detection of the MPT64 antigen test for the rapid confirmation of positive liquid cultures suggestive of MTB infection are commonly employed in clinical practice. However, our findings have revealed a higher rate of negative results among MTB isolates than previously reported, without confirmation of the lineage or sub-lineage of these strains. To further clarify this issue, we further validated the MPT64 antigen assay using isolates representing the major lineages and sub-lineages in this study.

## MATERIALS AND METHODS

### Isolate selection and culture

**Fig 1:**
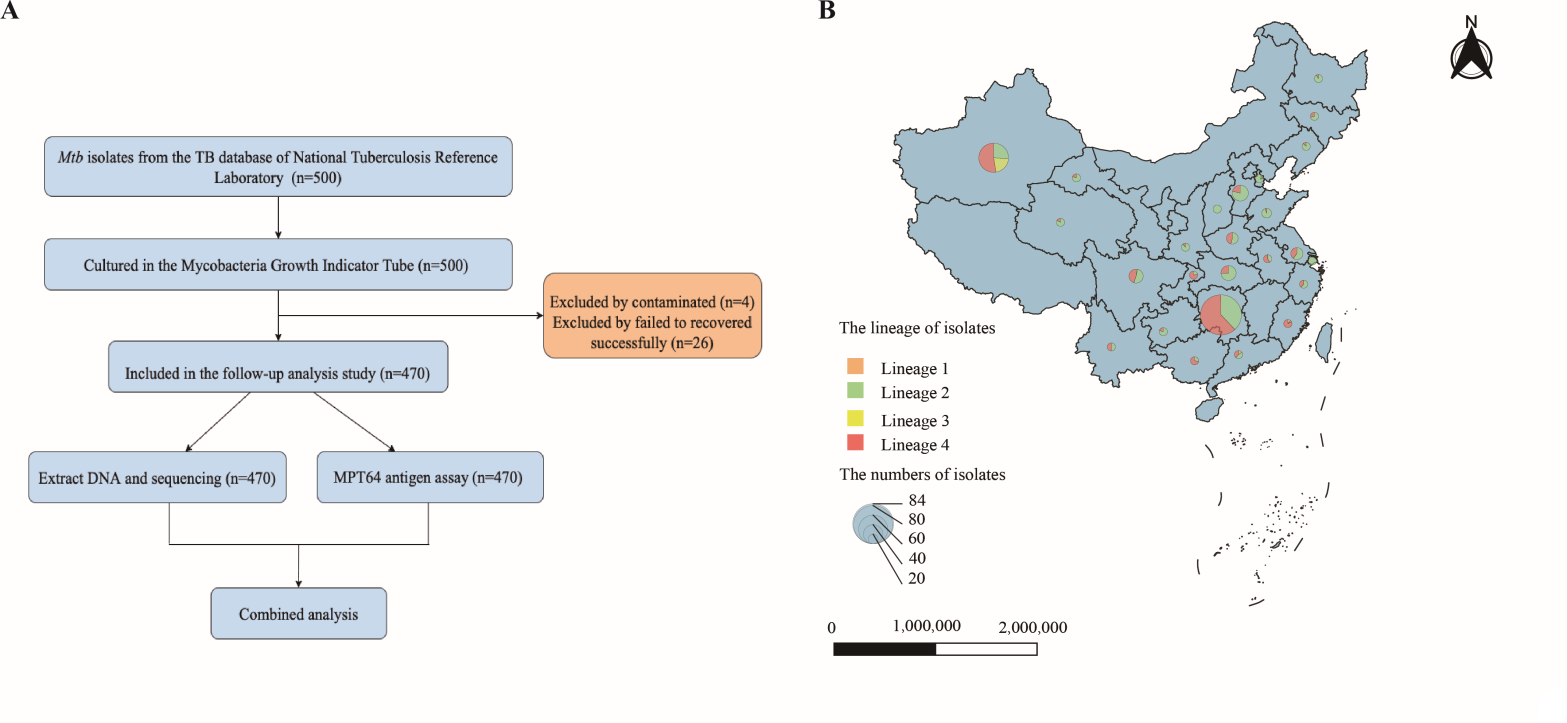
(**A**) Flow chart depicts the study design. (**B**) The geographic location and lineage of enrolled 470 *Mycobacterium tuberculosis* (MTB) isolates in China. The size of the pie charts reflected the number of isolates for each geolocation, and the pie charts displayed the distribution of isolates among the various lineages for each geolocation.

We randomly selected a total of 500 MTB strains isolated between 2013 and 2020 from the National Tuberculosis Reference Laboratory of China. These strains had been stored in a glycerol-containing storage medium at −80°C. According to the manufacturer’s instructions, the selected isolates were cultured in Mycobacteria Growth Indicator Tubes (MGIT; Becton, Dickinson and Company, Franklin Lakes, New Jersey, USA), utilizing the BACTEC MGIT 960 System (MGIT 960; Becton Dickinson Microbiology Systems, Sparks, Maryland, USA) at 37°C. Of these, four isolates were found to be contaminated, and an additional 26 isolates failed to recover successfully. Finally, a total of 470 isolates were included for subsequent analyses (Fig. 1A). These isolates were obtained from diverse geographical regions and contained various lineages (Lineages 1–4) (Fig. 1B). The geolocation and the number of isolates were plotted on a map generated using QGIS (v3.30).

### MPT64 antigen rapid identification test

The isolates were tested using the immunochemistry-based MPT64 antigen detection assay (Genesis, Hangzhou, China) according to the manufacturer’s instructions (12). The appearance of a colored signal line in both the test and control bands following a 15-min incubation period indicated a positive MPT64 test. Conversely, the appearance of a colored signal line only in the control band and not in the test band indicated a negative outcome (Fig. S1). To mitigate the potential influence of faint positive signals, MPT64 results were evaluated by a different observer who was blinded to the results of the first reading. The lineage analysis, blind MPT64 testing, and conventional identification of the isolates were all conducted by separate researchers.

### Whole-genome sequencing

DNA extraction from all isolates was performed using the cetyltrimethylammonium bromide method, as previously described (13). Subsequently, the DNA from each isolate was sequenced using the Illumina Hiseq X Ten (Illumina, Inc.) with 2 × 150 bp paired-end reads. All whole-genome sequencing procedures were conducted by Annoroad Gene Technology Company (Beijing, China).

### Bioinformatics analysis

Quality control of the sequence reads was performed using FastQC (v0.11.9). Following this, the reads were processed through the Clockwork pipeline with default parameters (v1.0). In the pipeline, human, nasopharyngeal flora, and human immunodeficiency virus-related reads were eliminated, and the remaining reads were trimmed (including adapters and low-quality ends) using Trimmomatic software and mapped to the reference genome MTB H37Rv (NC000962.3) with BWA-MEM. Genetic variants were independently called using Cortex and SAMtools, with the two call sets being merged to generate a final call set through Minos. Then, a multi-sequence.fasta alignment was generated from the merged.vcf file using vcf2phylip v2.0 (https://github.com/edgardomortiz/vcf2phylip/tree/v2.0), as previously reported (14). The phylogenetic tree was constructed using IQTree (v1.6.10) with automatic model selection and 1,000 bootstraps. The lineage and sub-lineage of isolates were identified based on the methodology of Coll et al. using the fast-lineage-caller v1.0 (https://github.com/farhat-lab/fast-lineage-caller) (15). The tertiary structure of the MPT64 protein was predicted using ColabFold v1.5.2 (AlphaFold2.ipynb) (16). The solution structure of the MPT64 protein was predicted using the previously solved structure (PDB accession no. 2HHI) as a reference.

### Bayesian skyline population

The aligned sequences of the isolates were analyzed using BEAST (v1.10.4) to construct a coalescent Bayesian skyline analysis with an uncorrelated lognormal distribution and an optimal evolution model of the GTR +G model (general time reversible with gamma-distributed rate variation, using four rate categories). Tracer (v1.7.2) was used for optimal model selection and population expansion estimation over time, and the ESS of the posterior distribution and all parameters exceeded 200.

## RESULTS

### Population structure of isolates

**Fig 2:**
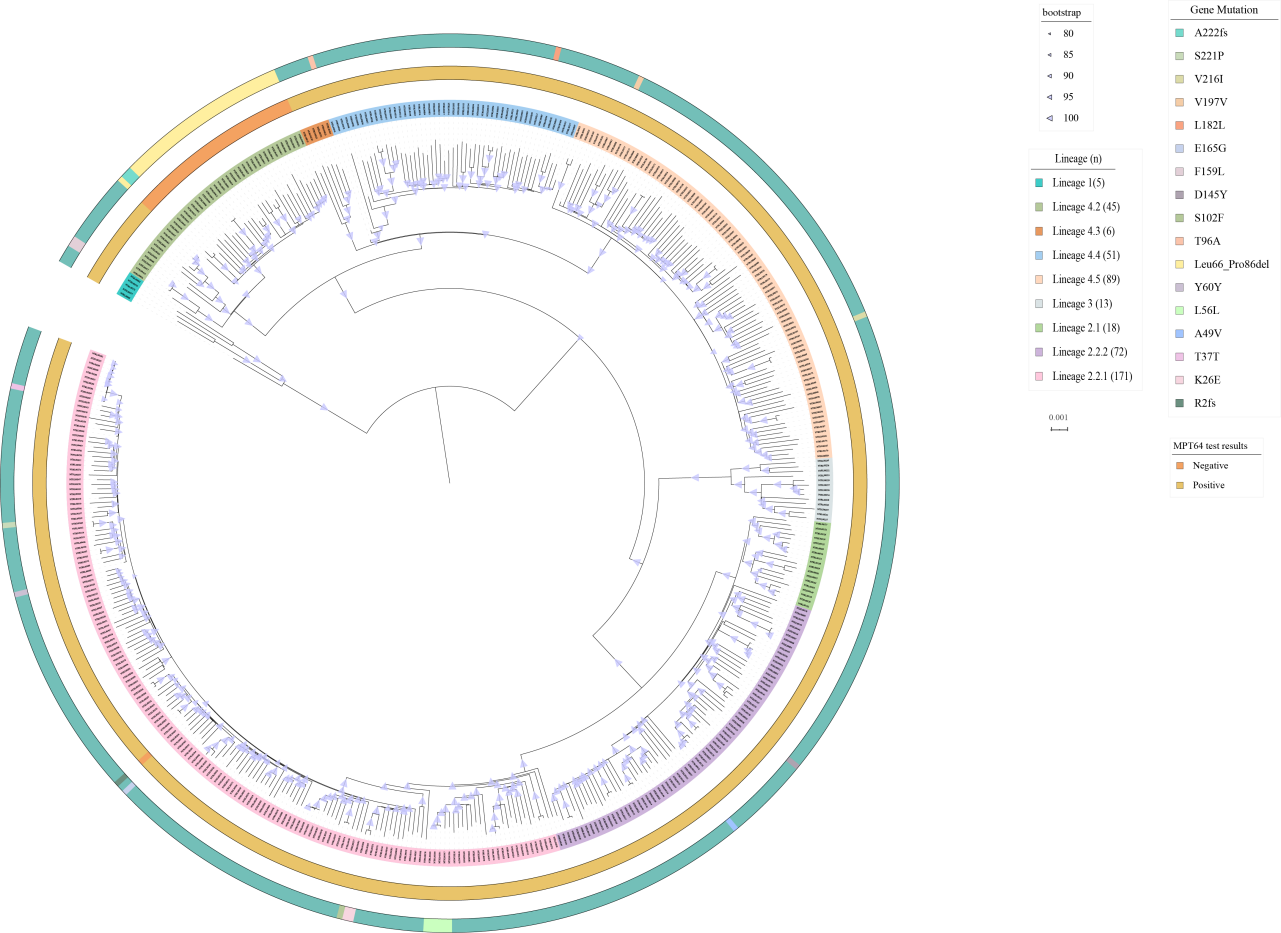
The maximum likelihood phylogenetic tree of 470 *Mycobacterium tuberculosis* (MTB) isolates was constructed by IQTREE with 1,000 bootstraps. The outer bands represent the lineage, MPT64 test results, and the *mpt64* gene mutation of the isolates (from inner to outer circles).

To elucidate the genetic diversity of the MTB isolates, we sequenced the isolates and constructed a phylogenetic tree for further genotyping (Fig. 2). Among these isolates, 261 (55.5%) belonged to Lineage 2 (L2) and 191 (40.6%) to Lineage 4 (L4), whereas 5 (1.1%) and 13 (2.8%) belonged to Lineage 1 (L1) and Lineage 3 (L3), respectively. Within L2, sub-lineages L2.2.1 and L2.2.2 accounted for 65.5% (171/261) and 27.6% (72/261), respectively. Among the L4 isolates in this study, four sub-lineages (L4.2, L4.3, L4.4, and L4.5) were identified. Most isolates belong to three sub-lineages (L4.2, L4.4, and L4.5), which were widely distributed and accounted for 96.7% of all L4 strains in China (17). Overall, these results indicate the high genetic diversity within our isolates, including the most prevalent sub-lineages in China (17).

### MPT64 antigen test results

We obtained MPT64 test results for all isolates with positive cultures, and the test positivity significantly varied among different sub-lineages (Fig. 2). All isolates from L1 and L3, as well as sub-lineages L2.1, L2.2.2, L4.3, L4.4, and L4.5, exhibited positive results in the MPT64 tests. The majority of isolates from sub-lineage L2.2.1 also yielded positive MPT64 test results, with only one isolate testing negative. However, in our study, only 26.7% (12/45) of isolates from sub-lineage L4.2 demonstrated a positive result for the MPT64 test, and all negative isolates (33/33, 100%) of L4.2 belonged to sub-lineage L4.2.2. These results demonstrated that the MPT64 antigen test has high sensitivity for sub-lineages L2.2.2, L4.3, L4.4, and L4.5 of MTB but exhibits low sensitivity for sub-lineage L4.2 of MTB.

### Genetic analysis of the *mpt64* gene

**Fig 3:**
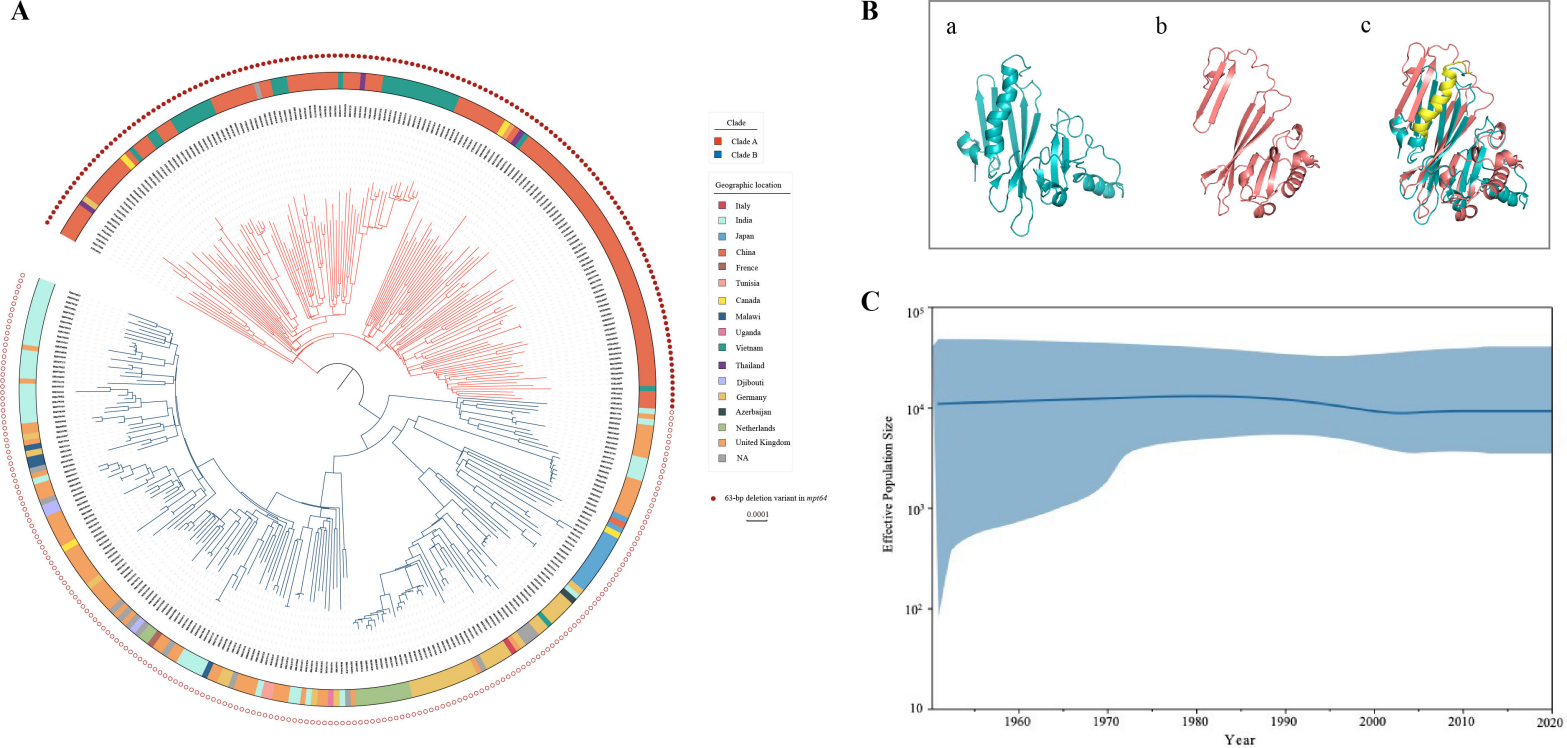
(**A**) The maximum likelihood phylogenetic tree of 342 L4.2.2 worldwide. The geographic location of isolates according to the color legend is shown on the tree. The red circle in the outer circle indicates that the isolates happened 63 bp deletion variants. (**B**) The tertiary structure of the MPT64 protein (**a**), MPT64 with 63 bp deletion (**b**), overlapped the two structures (**c**), and the yellow indicating the alpha helix lacked. (**C**) Coalescent Bayesian Skyline plots of the Clade A isolates using an uncorrelated lognormal relaxed clock model. The blue lines show the upper and lower bounds of the 95% HPD interval.

Our analysis of mutations in the *mpt64* gene revealed that the primary cause of the negative MPT64 antigen test among L4.2 isolates was the presence of 63 bp deletion (Fig. 2). Compared with the solution structure of Mpt64 (18), the tertiary structure of MPT64 with the 63 bp deletion lacked an alpha helix, resulting in a drastic change in the overall structural topology (Fig. 3B). This result is consistent with the previous report by Jiang et al. (19). Furthermore, two isolates (c.664delG) from L4.2 and one isolate (c.3delG) from L2.2.1 exhibited single-base deletion mutations, which led to frameshift variants, causing a substantial change in the overall structural topology. In addition, some isolates also harbored mutations that did not affect the outcome of the MPT64 antigen test. Among these isolates, two from L1 had the nonsynonymous mutation 477C > A (F159L). In L4.5, one isolate exhibited a nonsynonymous mutation 646G > A (V216I). Among the L2.2.2 isolates, two harbored nonsynonymous mutations 433G > T (D145Y) and 146C > T (A49V). Within L2.2.1, there were one, two, and three isolates with nonsynonymous mutations 661T > C (S221P), 305C > T (S102F), and 76A > G (K26E) and one, one, and five isolates with synonymous mutations 681G > A (L227L), 111C > G (T37T), and 166C > T (L56L), respectively.

### Deletion mutation of L4.2.2 MTB worldwide

To further understand the 63 bp deletion variant of the *mpt64* gene within the L4.2.2 population, we included and comparatively analyzed 342 isolates from various countries around the world (Table 1). A total of 270 publicly available genomes of L4.2.2 MTB strains from 16 countries worldwide were retrieved from the European Nucleotide Archive database (Table S1). The maximum likelihood phylogenetic tree of L4.2.2 exhibits two predominant and well-supported monophyletic lineages, referred to as Clades A and B (Fig. 3A). While isolates from different countries are distributed throughout the phylogenetic tree, those from Vietnam and China predominantly cluster within Clade A, whereas isolates from Europe belong to Clade B. Notably, we observed that all isolates within Clade A carried the 63 bp deletion variant in the *mpt64* gene, suggesting that these isolates independently evolved within the L4.2.2 lineage. Our Bayesian skyline analysis indicated that the effective population size of Clade A isolates has remained relatively constant (Fig. 3C). A recent study has suggested that China may serve as an intermediary location between Europe and Southeast Asia for L4.2.2 sub-lineages (20). However, further investigation is required to determine whether this is related to dormitory-specific evolution.

**TABLE 1:** The information of 342 L4.2.2 *Mycobacterium tuberculosis* used in this study

The country of the isolates	No. of the isolates		
	Clade A (*N* = 150)	Clade B (*N* = 192)	Total (*N* = 342)
China	111	1	112
United Kingdom	1	62	63
India	-	42	42
Germany	1	33	34
Vietnam	31	1	32
Japan	-	13	13
Netherlands	-	12	12
Malawi	-	4	4
Canada	2	2	4
Djibouti	-	3	3
Thailand	3	-	3
Tunisia	-	2	2
Azerbaijan	-	1	1
France	-	1	1
Italy	-	1	1
Uganda	-	1	1
NA	1	13	14

## DISCUSSION

Our study revealed an association between the 63 bp deletion variant in the *mpt64* gene and MTB L4.2.2 globally, suggesting that the MPT64 antigen assay demonstrates relatively lower performance in the rapid identification of MTB L4.2.2. Thus, there exists the risk of misclassifying certain L4.2.2 isolates as NTM in clinical settings when employing the MPT64 agent assay, leading to treatment delays.

Studies suggest that genetic polymorphisms in *mpt64* genes can introduce alterations in the MPT64 antigen, impacting MPT64 detection assays and resulting in false-negative outcomes. For example, Singh et al. found that changes in the MPT64 protein occurred after the 3rd, 9th, and 13th amino acids due to frameshift mutations, leading to negative results in the capilia test (11). Hillemann et al. demonstrated that frameshift mutations and premature stop codons, as well as the insertion of IS6110, could affect assay outcomes (21, 22). Qiu et al. reported on the impact of the 63 bp deletion and single-base mutations on TB diagnosis (23). In addition, the 63 bp deletion variant has been reported in a set of clinical strains (19, 24), but was not previously associated with the lineage of the isolates. To our knowledge, we are the first to identify and report a 63 bp variant explicitly associated with the L4.2.2 sub-lineages globally. Additionally, some nonsynonymous and synonymous mutations in the *mpt64* gene have been reported (10, 25), which may have limited effects on protein structure and rarely affect the diagnosis through MPT64 detection assays.

Although it has been demonstrated that the MPT64 antigen test exhibits certain lineage-specific limitations (L5 and L6 of MTBC isolates), these limitations are associated with isolates that have a restricted regional distribution (5, 15). In contrast, L4.2 isolates are widely distributed globally, presenting a diagnostic challenge for MPT64-based tests. Previous research on the geographical and global distribution of L4 has revealed that L4.2 is prevalent in high proportions among isolates from specific countries in Asia and Africa and accounts for a certain percentage in Europe (26). According to a recent study, three sub-lineages (L4.2.2, L4.4.2, and L4.5) constitute 94%, 81%, 51%, and 9% of L4 in China, Thailand, Vietnam, and Indonesia, respectively (20).

Our study showed that isolates containing the 63 bp deletion variant belong to Clade A within the L4.2.2 lineage. This suggests that in countries where Clade A isolates are prevalent, such as China and Vietnam, MPT64-negative test results should be confirmed using alternative identification techniques before classifying them as NTM. To address the limitations of currently available MPT64-based immunochromatographic tests, more refined approaches for TB diagnosis, such as the use of recombinant antibodies (27), can be developed. Furthermore, further investigation is needed to determine whether the 63 bp deletion variant in MPT64 affects the pathogenicity of the isolates and their clinical symptoms.

Several limitations exist in our study. First, this study used only one immunochemistry-based MPT64 antigen detection assay (Genesis, Hangzhou, China). However, our whole-genome sequencing analysis of the isolates helped compensate for potential errors associated with this method to some extent. Second, we were unable to accurately infer the precise differentiation time of L4.2.2 sub-lineages, such as Clades A and B, due to incomplete information regarding the isolation time of some isolates. Further research is required to address this aspect.

### Conclusion

To our knowledge, this is the first report on the association of a 63 bp deletion variant in the *mpt64* gene with L4.2.2 MTB globally, and it highlights that MPT64 immunochromogenic tests should be cautiously used, especially in regions where L4.2.2 isolates are common, such as China and Vietnam.

## Data Availability

The accession numbers of the isolates were shown in Table S1.

## SUPPLEMENTAL MATERIAL

Fig. S1 spectrum.01842-23-s0001.pdf MPT64 antigen test with a positive and a negative result.

Table S1 spectrum.01842-23-s0002.xlsx Information and sequences of 270 L4.2.2 isolates from 16 countries worldwide downloaded from the European Nucleotide Archive (ENA) database.

ASM does not own the copyrights to Supplemental Material that may be linked to, or accessed through, an article. The authors have granted ASM a non-exclusive, world-wide license to publish the Supplemental Material files. Please contact the corresponding author directly for reuse.
