# Supplementary figures and images for: Association of lineage 4.2.2 of Mycobacterium tuberculosis with the 63-bp deletion variant of the mpt64 gene
Source: Microbiol Spectr. 2023 Nov 10;11(6):e01842-23. doi: 10.1128/spectrum.01842-23 (PMC10714870; doi:10.1128/spectrum.01842-23)

Postivte

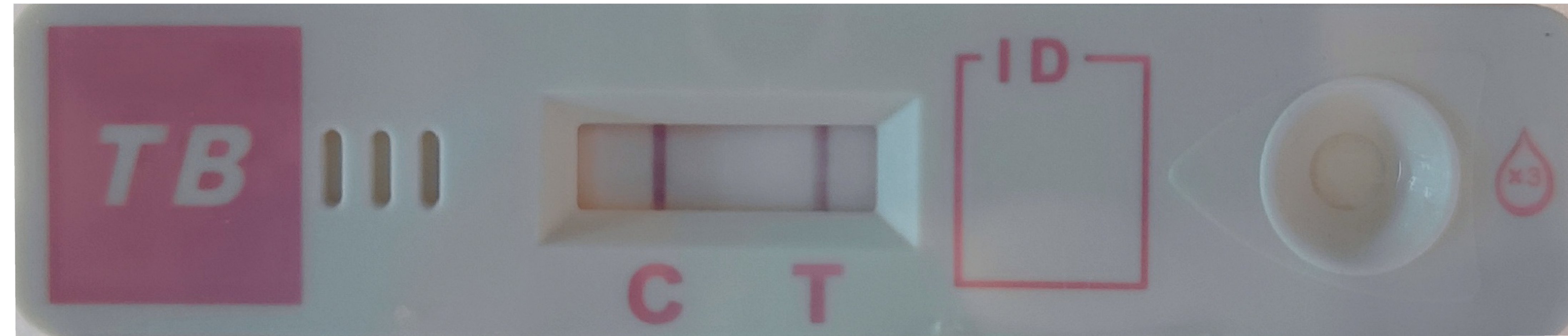

Negative

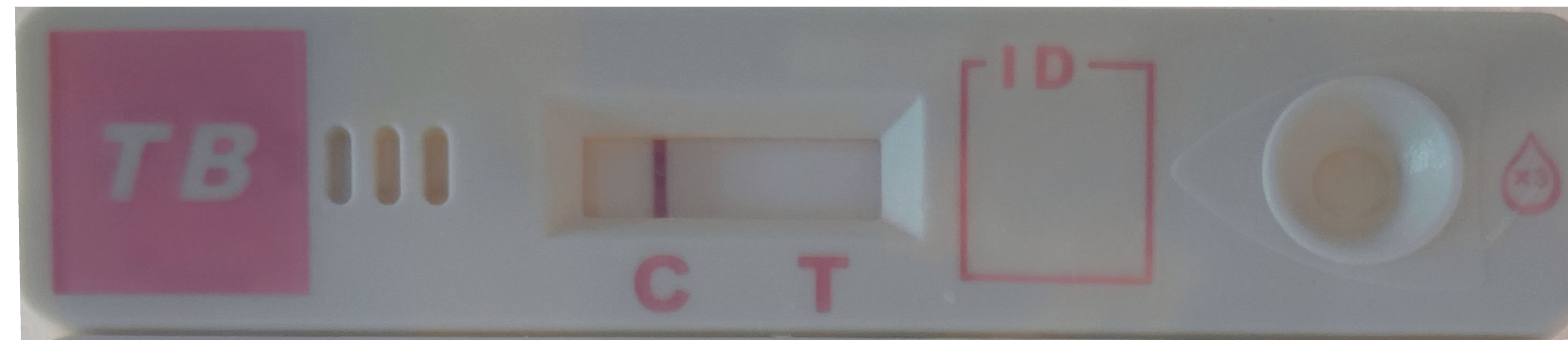

Figure S1. The figure of the MPT64 antigen test with a positive and negative.

Supplement: Fig. S1 — MPT64 antigen test with a positive and a negative result. [file spectrum.01842-23-s0001.pdf]
